# Wnt Signaling in Neurogenesis during Aging and Physical Activity

**DOI:** 10.3390/brainsci2040745

**Published:** 2012-12-14

**Authors:** Michael Chen, Huong Do

**Affiliations:** Department of Biological Sciences, California State University, 5151 State University Drive, Los Angeles, CA 90032, USA; E-Mail: meofomi@gmail.com

**Keywords:** Wnt, running, exercise, neurogenesis, aging, hippocampus

## Abstract

Over the past decade, much progress has been made regarding our understanding of neurogenesis in both young and old animals and where it occurs throughout the lifespan, although the growth of new neurons declines with increasing age. In addition, physical activity can reverse this age-dependent decline in neurogenesis. Highly correlated with this decline is the degree of inter and intracellular Wnt signaling, the molecular mechanisms of which have only recently started to be elucidated. So far, most of what we know about intracellular signaling during/following exercise centers around the CREB/CRE initiated transcriptional events. Relatively little is known, however, about how aging and physical activity affect the Wnt signaling pathway. Herein, we briefly review the salient features of neurogenesis in young and then in old adult animals. Then, we discuss Wnt signaling and review the very few *in vitro* and *in vivo* studies that have examined the Wnt signaling pathways in aging and physical activity.

## 1. Introduction

The myriad benefits physical activity confers is now universally accepted, not only on the general health of the individual, but also on brain function [1,2,3,4]. Physical activity has been shown to enhance memory and cognition in both humans [5,6,7] and other animals [8,9,10,11,12]. As long as exercise is performed consistently, these benefits are robust and enduring [8,13,14], even if such exercise is started relatively late in life [15,16]. The study of such mechanisms and pathways reveals not only how exercise benefits the brain, but also underscores the need to elucidate natural progression of how neural circuitry develops and refines itself over the lifespan in response to the varying environmental demands placed on the organism. Thus, the inter and intracellular signaling pathways and plasticity of the brain of a habitually physically active animal (a normal and natural lifestyle) will be significantly different from one who is confined in a cage with little-to-no opportunity to exercise (a life of impoverishment and deprivation) [17,18].

One of the enduring neuronal hallmarks of an active and enriching lifestyle is an enhanced ability of the brain to grow new neurons or adult neurogenesis [19]—that is, more new neurons than would appear in a sedentary lifestyle. Over the past several years, there has been intense interest and effort focusing on the intracellular signaling pathways in the hippocampus, one of two putative neurogenic structures and which is well known for its central role in learning and memory, particularly spatial memory [20]. As such, decreased intracellular MAPK and Akt pathway signaling [21], decreased intercellular neural cell adhesion molecule [22] and altered neuronal circuitry, such as dendritic spine shortening [23,24], have also been shown in the hippocampus to occur in various disorders [25], such as depression [2,26,27] and Alzheimer’s Disease [28,29,30]. These molecular and cellular aberrations may underlie the behavioral and clinical manifestations of these disorders. Thus, an inability to learn new coping skills, or forget old ones, in response to life-changing events, such as that which may occur with stress and depression, may be a hippocampally derived problem [22,31,32]. Such behavioral or learning problems may belie an inability of the hippocampus to adequately grow new neurons in response to the stressors of environmentally imposed demands [24].

One signaling pathway that is putatively known to regulate neurogenesis is the canonical Wnt or Wnt/β-catenin signaling pathway [33]. Although this pathway has been characterized in depth and is the subject of several excellent reviews [33,34,35], relatively few studies have addressed how this pathway responds to physical activity and aging. The purpose of this brief review, therefore, will be to elucidate what we currently know about hippocampal Wnt/β-catenin signaling, induced neurogenesis during aging and the effects of physical activity on this process.

## 2. Wnt Signaling

Wnts are a family of ligand proteins whose numbers exceed 14 and whose receptors, termed frizzled (fzl), exceed at least eight in mammals [36]. Although this large number of ligands and receptors allow the mammalian genome to regulate crucial central nervous system functions, such as development, each Wnt isoform/receptor combination may act like a molecular switch by activating certain transcription factors as they interact with their specific DNA elements (see below).

**Figure 1:**
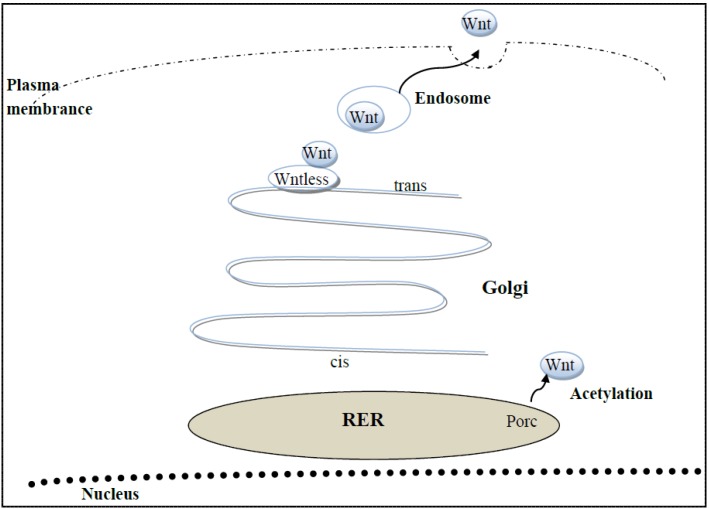
Wnts are secreted by astrocytes, neural stem cells or neural progenitor cells. Wnts are transcribed and translated and then acetylated by the acetyltransferase, Porcupine (Porc), in the endoplasmic reticulum. On the trans face of the Golgi, is a multimeric protein, called wntless, which aids in the transfer of Wnts to endosomes. Upon fusion of the two membranes, Wnt is secreted to the exterior of the cell.

Wnts are postranslationally acetylated in the endoplasmic reticulum by an acetyltransferase, called porcupine (Figure 1). Following transfer and subsequent removal from the Golgi, another protein, called Wntless, acts like a chaperone for the transfer of Wnts to endosomes, whose membrane fuses with the plasma membrane, resulting in the secretion of Wnts to the extracellular space [37], Figure 1. What follows is a brief review highlighting the three Wnts that have been shown to play the largest roles in adult hippocampal neurogenesis: Wnt3(a), Wnt1 and Wnt7(a).

### 2.1. Wnt3(a)

There is more evidence for the importance of Wnt3(a) in adult hippocampal neurogenesis than for any other Wnts. Wnt3 is expressed by adult hippocampal stem and progenitor cells [38] and astrocytes [33] and is an intrinsic regulator of hippocampal neurogenesis by modulating the generation of newborn granule cells in the dentate gyrus [38]. Conversely, lentiviral expression of a dominant negative mutant in the dentate gyrus has led to decreased hippocampal neurogenesis and decreased long-term retention of spatial and object recognition memories in adult rats [39]. Wnts secreted by adult hippocampal progenitors self-stimulate [40] canonical Wnt signaling, but inhibition of this autocrine Wnt pathway results in an increased number of neurons formed and concomitant loss of multipotency among progenitors [41].

*In vitro*, there is much evidence for the significance of Wnt3 as a central player in adult hippocampal neurogenesis. Wnt3 has been shown to be required for the generation of new granule cells: Wnt3 mutation has led to decreased hippocampal neurogenesis [42] and application of Wnt3(a) resulted in adult hippocampal neurogenesis [41]. In addition, Wnt3a application to cultured neural stem cells promoted their differentiation, but inhibited their maintenance, suggested by the increased number of astrocytes and increased differentiation into MAP-2-positive neurons [43]. This is consistent with more recent findings that Wnt3a application accelerates the transition from neural progenitor cell to differentiated granule cell by shortening the duration of the cell cycle of the former by nearly three hours [44]. Moreover, Wnt3a has a crucial role in hippocampal development as targeted deletion of this Wnt leads to the lack of a hippocampus and dentate gyrus [45]. Finally, application or exposure to Wnt3(a) has increased expression of various neuronal markers, such as doublecortin (dcx, a microtubule-binding protein; see below) in proliferating neuroblasts and immature postmitotic neurons in the dentate gyrus (reviewed by Gage [33] dishevelled2 (a fzl receptor binding protein; see below) in an immortalized human neuroprogenitor cell line [46], NeuroD1 (a transcription factor in the Wnt canonical pathway; see below), and nuclear and cytoplasmic β-catenin in synaptogenesis [47].

Finally, and importantly for signaling interaction and receptor cross-talk with several other growth-promoting pathways, this indicates that Wnt3 signaling plays an important role along with others, such as sonic hedgehog [48], brain-derived neurotrophic factor (BDNF) [49], vascular endothelial growth factor [50] and insulin [51] in adult hippocampal neurogenesis. Consistently, Wnt3a (and Wnt7a, see below) also promotes presynaptic protein clustering, increased presynaptic recycling sites and increased rate of synaptic vesicle neurotransmitter release [52,53].

### 2.2. Wnt1

Although cells expressing Wnt1 makes up a relatively small percentage of hippocampal stem cells that express Wnt proteins (only about 10%, [54]), Wnt1 has also been shown to be required for neurogenesis in the subgranular zone as a dominant negative mutant of Wnt1 blocks this process [42]. More recent evidence indicates that mutation of Wnt1 results in the absence of the midbrain (reviewed in [55]).

### 2.3. Wnt7

Environmental enrichment increases expression of Wnt7(a) in CA3 pyramidal neurons [56]; conversely, application of Wnt7 to these neurons mimicked the effects of environmental enrichment on synapse and mossy fiber terminal Wnt7 levels whose concentrations reached their peak in mice aged 6–12 months; the age-related decline in synapses and Wnt7 were reversed by environmental enrichment [56]. Again, consistently, just as with Wnt3 (above), Wnt7 also increases presynaptic protein clustering, vesicle aggregation and neurotransmitter release [52,53] and synaptogenesis through up-regulation of the fzl5 receptor [57,58] and nuclear and cytoplasmic β-catenin levels [47].

*In vitro* application of Wnt7(a) to adult hippocampal neuroprogenitors leads to increased proliferation [41], whereas mutation of Wnt7a results in decreased hippocampal neurogenesis (reviewed in [55]). Just as with Wnt3 (above), *in vitro* stimulation of an immortalized human neuroprogenitor cell line resulted in increased Wnt7a mRNA and a corresponding up-regulation of fzl7 and fzl9 receptor transcripts [46]. 

### 2.4. Wnt Signaling Receptors and Cascades

Wnt signaling is turned on when Wnts secreted from either an astrocyte or a neuroprogenitor cell diffuses to a nearby neuroprogenitor cell (paracrine), where it finds its receptor, fzl, or it may bind to fzl embedded in the membrane of the same cell that secreted it (autocrine). Frizzled is a G-protein-coupled receptor (GPCR) whose extracellular *N*-terminus contains a cysteine-rich domain that directly binds Wnt [37]. The *C*-terminus has a conserved KTxxxW motif that interacts with disheveled (dsh1) through its PDZ domain [37]. Besides signaling through dsh1, fzl may also activate intracellular signaling through heterotrimeric G-proteins [37].

**Figure 2:**
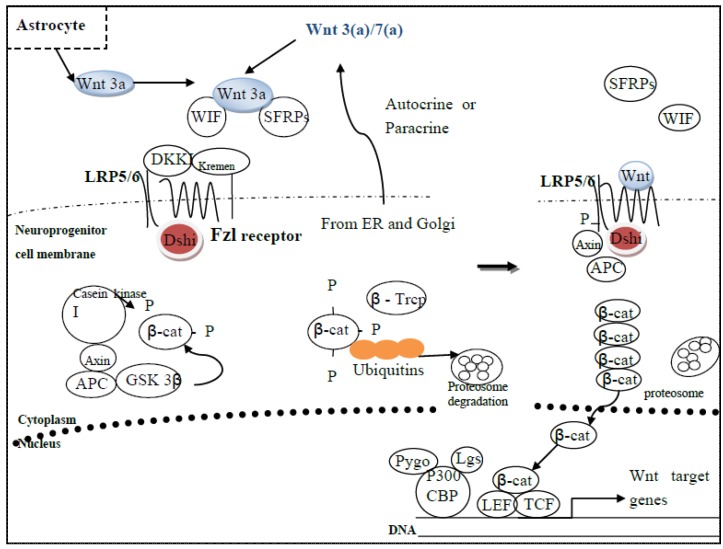
(Left) Wnt signaling is turned off when the ligand, although secreted by nearby astrocytes or neuroprogenitor cells, is bound by various Wnt inhibitors, such as Wnt inhibitory factor (WIF) and secreted fzl-related proteins (SFRPs). The fzl receptor remains bound by disheveled (dsh1) and LRP5/6 is bound by Dickopf (DKK1)-kremen complex, which helps anchor the complex into the membrane. With adenomatous polyposis (APC) and axin bound to casein kinase 1 and GSK-3β, these two kinases can now phosphorylate β-catenin, which is then sequestered by ubiquitins with the help of β-Trcp. β-Catenin is then degraded in the proteosome. (Right) Wnt signaling is turned on when Wnt binds fzl and the LRP5/6 co-receptor, promoting axin to dissociate from APC and dsh1 to phosphorylate LRP5/6. Meanwhile, casein kinase 1 phosphorylates GSK-3β, thereby inactivating it. β-Catenin then accumulates in the cytoplasm, enters the nucleus, where it binds to TCF/LEF and co-activators, such as pygopus (Pygo), legless (lgs), and P300/CBP, leading to the transcription of Wnt target genes.

Low-density lipoprotein receptor-related protein 5/6 (LRP 5/6) are co-receptors of fzl and are critical for Wnt signaling, as deletion mutants of this co-receptor completely abolishes Wnt signaling [37]. Wnt binding causes a conformational change in fzl, resulting in recruitment of dsh1 to the cytoplasmic tail of the receptor through its intracellular KTxxxW motif [37]. Wnt also causes the phosphorylation of dsh1 through activation of, perhaps, casein kinase 1, as well as of LRP5/6 whose C-terminus then reacts with axin, which is a downstream inhibitory component of the Wnt cascade. As axin is mobilized to the cell membrane, its activity is inhibited, resulting in the stabilization of β-catenin (Figure 2). Additionally, Wnt binding to fzl induces the phosphorylation of LRP 5/6 at two sites, one of which creates a docking site for axin, while the other is phosphorylated at the *N*-terminus by casein kinase 1 [37].

It is well known that to enter the nucleus, β-catenin has a nuclear export sequence, which explains its ability to enter and exit the nucleus in response to the status of Wnt signaling. Once in the nucleus, β-catenin interacts with the TCF family of transcription factors, which includes TCF-1, LEF-1, TCF-3, and TCF-4. When unbound, TCF/LEF family members actively recruit co-repressors histone deacetylases (HDACs) and GROUCHO/TLE-1 to inhibit transcription. GROUCHO/TLE, in turn, interacts with hypoacetylated histone H3, perhaps to maintain the structural integrity of the chromatin [37]. However, once β-catenin enters the nucleus, it binds TCF-4, displaces GROUCHO/TLE-1 from TCF/LEF, and then recruits co-activators through its *N*- and *C*-terminal transactivation domains [37]. The *N*-terminal transactivation domain of β-catenin interacts directly with BCL9/legless (Lgs), which then recruits the transcriptional co-activator, Pygopus (Pygo) [37]. The *C*-terminal transactivation domain of β-catenin recruits the histone acetylators, P300 and CBP, thereby loosening the chromatin structure and facilitating the binding of other transcriptional co-activators ([37] and references cited therein) (Figure 2).

Wnt signaling is turned off when the ligand is bound in the extracellular space by one or more inhibitor and intracellular β-catenin is subsequently degraded (Figure 2). The clear role of canonical Wnt signaling is to regulate the stability of β-catenin whose cytoplasmic concentrations is tightly regulated by the ubiquitin-proteosome degradation complex, which contains the scaffold protein, axin, as well as β-catenin, casein kinase 1, glycogen synthease kinase-3β (GSK3β), and tumor suppressor protein adenomatous polyposis (APC) ([37] and references cited therein). Phosphorylated APC displaces β-catenin from the axin complex because it has a higher affinity for the former. After β-catenin is phosphorylated at the *N*-terminus by casein kinase 1 and GSK-3β, it is then ubiquitinated by β-Trcp, upon which, β-catenin is immediately degraded by the proteosome (Figure 2).

β-Catenin regulates the basic helix-loop-helix transcription factor, NeuroD1, which is required for the survival and differentiation of newborn neurons in the adult subgranular zone. A wide variety of stimuli (e.g., running, seizures, environmental enrichment) can profoundly induce neurogenesis. It is possible that these stimuli may act, in part, via NeuroD1 target downstream genes to control the survival and maturation of newborn neurons [59]. β-Catenin also associates with LEF/TCF binding sites in the Prox1 enhancer and promotes Prox1 expression in adult hippocampal neural stem cells. 

Prox1 is expressed in neural progenitors and in both mature and immature neurons in the adult dentate gyrus, indicating that Prox1 is a direct Wnt target that promotes neurogenesis [60] by regulating the expression of differentiation and survival factors that are required for early and late stages of hippocampal neurogenesis [60]. Thus, β-catenin-TCF/LEF-dependent transcription selectively up-regulates Prox1 expression, leading to the expression of VEGF receptor, FGF receptor and α-9 integrin [60]. Once a granule cell has fully matured, therefore, Prox1 expression levels remain high, rather than being down-regulated [60].

Both NeuroD1 and Prox1 are also regulated. The Sox family of transcription factors represses expression of prosurvival genes. Specifically, Sox2/9 maintains neural stem cells in an undifferentiated state [60]. Conversely, the Prox1 enhancer region represses Sox9 expression [60]. Thus, in the mouse dentate gyrus, Wnt signaling and repressed Sox2 lead to increased NeuroD1 expression [61]. Further, Wnt3 knockouts and NeuroD1 deficiency led to no dentate gyrus formation [61]. Thus, Wnt/β-catenin signaling contributes to the gradual progression of adult hippocampal neurogenesis by removing Sox2 repression and turning on NeuroD1 [61] and Prox1 [60].

Repression of the NeuroD1 gene, and therefore, neurogenesis, is carried out by the repression complex, HDAC/Sox2, which binds to the Sox/LEF element, which is located in the NeuroD1 promotor [62]. Repression of NeuroD1 also prevents transcription of genes at any number of LINE1 (L1) retrotransposon loci. Astocytic secretion of Wnt3a turns the Sox/LEF switch on via β-catenin activation, which accumulates in the neural progenitor cell nucleus, where it complexes with and activates the TCF/LEF. This leads to transcription of the NeuroD1 gene, which allows granule cell neurogenesis and maturation. Moreover, the L1 family of mobile transposable elements are up-regulated and retrotransposed during neurogenesis. For example, one particular L1 element could be up-regulated by Wnt/TCF signaling through the same direct interaction as the NeuroD1 gene. Several of these LINE1 elements are located near other genes that are involved in neurogenesis, such as dcx and neuregulin4 [62]. Because L1 retro-element sequences contain Sox/LEF DNA regulatory elements, the Sox/LEF binding sites induce promotors to cause nearby neuronal genes to become de-silenced and activated during adult neurogenesis. Because L1 elements are active during neurogenesis, both NeuroD1 and LINE1 transcription factor expression are specifically induced only when Sox2-positive neural stem cells transition to newborn neurons upon Wnt/β-catenin activation [61].

## 3. Stages of Adult Hippocampal Neurogenesis

Several years ago, Kempermann *et al.* [63] outlined six clearly identifiable adult hippocampal neurogenic stages, based on morphology, the ability to proliferate and the expression of various markers, such as glial fibrillary acidic protein (GFAP), Sox2, doublecortin (dcx), calretinin, calbindin and NeuN. Stage 1 begins the putative stem cell stage (type I cells) in the dentate gyrus subgranular zone wherein the stem cell has virtually unlimited renewal capacity; morphologically, this cell has processes reminiscent of both astrocytes and radial glial cells. Both nestin and GFAP are therefore expressed at this stage. In the subgranular zone, there are three populations of precursors that will eventually become mature granule cells: radial neural stem cells (type I progenitors, above), nonradial neural stem cells (type 2 progenitors) and neuroblasts. Neuroblasts will migrate into the adjacent granule cell layer where they will mature into granule neurons [64].

At Stage 2, (type 2a cells), the transition to putative progenitor cell with limited self-renewal capacity has been made, but GFAP is no longer expressed. At Stages 3 and 4, (types 2b and 3), the progenitor cell still has limited self-renewal capacity. As progression from Stages 2 through 4 is made, the lineage becomes increasingly determined to that of a neuron. This is an important milestone, because sometime between Stages 3 and 4, glial markers are no longer expressed. Thus, no overlap between glial and neuronal markers has ever been observed at this time [63]. After Stage 3, (type 2b cells), nestin is no longer expressed; and after Stage 2, dcx expression begins (type 2b cells). Doublecortin is a microtubule-binding protein that is expressed by at least some proliferating neuroblasts and immature postmitotic neurons in the adult dentate gyrus. [33].

Then, the transition from Stage 4 (type 3 cells) to Stage 5 (immature granule cell) marks the end of the mitotic phase and begins the postmitotic phase. During Stage 4 in type 3 cells, the nucleus enlarges and the expression of neural cell adhesion molecules begins [63]. At Stage 5, during the early postmitotic period, as dcx expression persists and calretinin and NeuN expression begins, the neuron starts sending out connections to establish itself in the network, beginning the selection and expression of genes that would help ensure its long-term survival. Finally, by Stage 6 (postmitotic granule cell), both dcx and calretinin expression have ceased, but that of calbindin has begun; NeuN expression persists. At this final stage, the terminally differentiated granule cell elaborates its processes, weaving itself into the hippocampal circuitry [63].

## 4. Physical Activity Increases Neurogenesis in Young Adults

It is well known that in the young adult rat, the dentate gyrus generates thousands of new cells daily [65], although clear estimates of how many thousands of cells are not currently known. Given that BrdU labeling/incorporation is at best 50%, conservative estimates of new granule cells during one month in the life of a young adult is 5% [65]. Later, Cameron and McKay [66] found that BrdU-labeled newly born cells appeared in the inner granule cell layer. In the young adult rat, up to 9000 new neurons are born per day and survive with a half-life of 28 days [66]. During adulthood, the numbers of these granule cells decrease with age [65,67], suggesting that over the lifetime of a rat, the hippocampus would grow gradually larger, were it not, however, for substantial granule cell death [68], via exercise-induced accelerated granule cell turnover in the dentate gyrus [69], leading to a rather static population of neurons with death rate keeping up with birth rate. Thus, it is possible that although exercise has been shown to increase hippocampal volume in older humans [70,71], it is probably not due to neurogenesis [69], but rather, to any number of lifestyle and health related issues (see [70] for brief review).

Physical activity increases vascularization throughout the frontal lobe and hippocampus, resulting in increased oxygen delivery [72] and neuronal survival and neurogenesis [72]; and learning further enhances the survival of these neurons [73,74]. Running has also been shown to increase neurogenesis and dendritic complexity and length of granule cell processes [75] in the dentate gyrus [65,75], regardless of whether they are wild- or captive-bred rats [76]: These investigators hypothesized that the extent of neurogenesis would be higher in wild rats than in the captive-bred strains, but instead, found no significant differences in neurogenesis between the two groups, suggesting that the highly stimulating enriched environment of the former did not influence neurogenesis [76]. In addition, Hauser *et al.* [77] did not find any two-week running effect on neurogenesis in the hippocampi of wild-caught mice, compared to that in wild-caught sedentary mice. Both of these findings flew in the face of earlier, well-established results [19,78,79,80]. The caveat here, however, is that strain differences might account for the neurogenic potential of these two groups. Moreover, besides differences in strain [81], differences in hippocampal neurogenic potential may be influenced by motivation and emotional content of stimuli [82] and species lifespan [83]. These studies underscore the fact that rodents appear to differ with respect to their hippocampal anatomy. For example, the neurogenesis quiescent zone (NQZ) does not seem to exist in mice. The NQZ is a small region in the rat dentate gyrus in which neurogenesis does not occur and until adolescence, does not reveal any mitotic neurons, but which can be activated after only one week of exercise [84]. It is not known why this difference exists between rats and mice [84], but it is possible that in the mouse, age-induced neurotrophic support occurs evenly throughout the entire length of the dentate gyrus, rather than in an exclusive highly focused sub-region of the dentate gyrus [84]. It is also possible that in rats, dentate gyrus granule cells are more numerous and mature faster than in that of mice [85]. After two months of running exercise, neurogenesis is significantly increased in the mouse dentate gyrus [86]. The importance of running as the critical factor of enriching lifestyle in stimulating significant neurogenesis has been demonstrated by several investigators. The stimulating complexity of an enriched environment is not enough to increase brain-derived neurotrophic factor (BDNF) levels and neurogenesis if running *per se* is not part of that environment [87]. Likewise, exercise can prime the neurogenic niche for possible eventual environmental enrichment [79]. Consistently, Steiner *et al*. [88] had earlier found that running exercise, but not in an enriched environment, induced astrogenesis, which is critical for Wnt signaling (below). Further, these investigators [88] provide evidence delineated by Kempermann *et al.* [63] (above) that the adult dentate gyrus has two distinct populations of cells, glia and neurons, that do not overlap at any neurogenic stage [63,88].

### Intracellular Signaling of Neurogeneis in Young Adults

**Figure 3:**
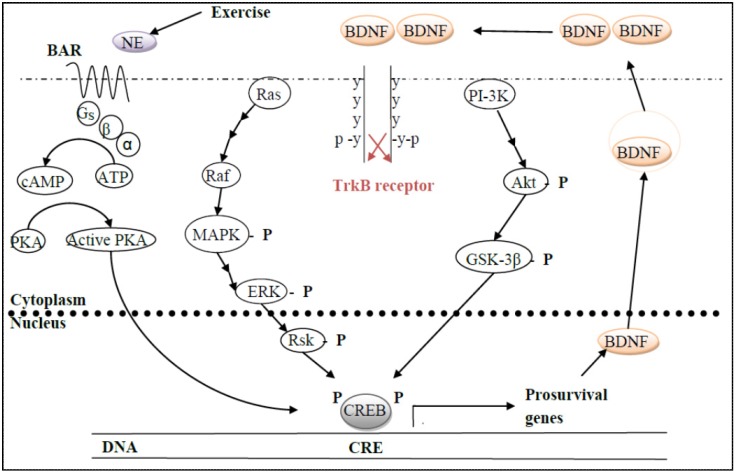
Exercise releases norepinephrine (NE), which binds its GPCR, β-adrenergic receptor (βAR), and then activates cAMP-dependent protein kinase A (PKA), which is only one of many kinases capable of phosphorylating the transcription factor, cAMP-response element binding protein (CREB). Activated CREB is then able to transcribe a wide array of pro-survival genes, one of which is BDNF. The neurotrophin is then packaged into vesicles and released to the extracellular space, where it dimerizes before binding to its receptor, TrkB, which also dimerizes upon ligand binding. TrkB dimerization results in the receptor cross-phosphorylating on opposite tyrosine residues, which then activates many different downstream intracellular signaling pro-survival pathways, only two of which are illustrated here: the phosphatidylinositol 3′-kinase (PI-3K) and mitogen-activated protein kinase (MAPK), ultimately also phosphorylating CREB for continued transcription of BDNF. Any of these pathways can be slowed down or inactivated by the action of phosphatases.

Adult hippocampal neurogenesis enhances learning [89] and prevents cognitive decline [90] through an up-regulation of BDNF, which, upon binding to its receptor, TrkB [90], activates a wide array of intracellular signaling cell survival pathways (see [91] for review; [90], (Figure 3)). Likewise, exercise in young adults up-regulates various neurotrophins, particularly BDNF (and TrkB), in the hippocampus [87,91,92,93], eNOS and NO, leading to enhanced angiogenesis [94,95] or insulin-like growth factor I (IGF-1) from the periphery [96], thereby promoting neurogenesis [97,98]. As an epigenetic mechanism, such up-regulation may be mediated via suspension of the transcriptional repressing effects on transcription, specifically, for example, methyl CpG binding protein 2 (MeCp2), which is most commonly seen in sedentary rats (reviewed in [92]). As neurons depolarize McCp2 dissociates from the *bdnf* promotor IV region and then is phosphorylated as much as 25% in adult exercising rats [92].

Exercise-induced plasticity is mediated by hippocampal BDNF and myriad genes involved in synaptic plasticity (see [91] for review). The locus coeruleus, which synthesizes and releases norepinephrine, and the raphé, which synthesizes and releases serotonin, sends afferents to the hippocampus, which, in turn releases BDNF, thereby activating other plasticity-related genes, including those involved in neurogenesis [99,100], Figure 3. In addition, the septal nucleus, which synthesizes and releases acetylcholine, is also activated in response to exercise, which, in turn, promoted neurogenesis; lesioning this system decreased neurogenesis and application of cholinergic drugs promoted it in both young and aged mice [101].

## 5. Physical Activity Partially Reverses the Age-Related Decline in Neurogenesis

There is much evidence that the mental health benefits derived from physical activity, particularly in old age, is mediated by an up-regulation of neurotrophic factors [2,28,102,103]. One of the critical cell survival pathways downstream from that of BDNF-TrkB binding is that of PI-3K/Akt (Figure 3), which has been shown to be activated following two weeks of voluntary wheel running [104]. Consistently, running also increases the survival of newly-generated dentate gyrus neurons via this pathway, as well as activating or inducing anti-apoptotic proteins [105], but without affecting the ERK pathway [106]. Although hippocampal neurogenesis is significantly decreased in the aging brain, compared to that in younger animals, the newly added neurons seem to be functionally similar (reviewed in [107]), suggesting that neurogenesis in the aged brain is simply down-regulated, rather than pathological [107]. Indeed, over 1100 genes have been identified that are differentially expressed as a result of aging between the young and old dentate gyrus [108]. Because exercise promotes neurogenesis, which, in turn, enhances learning/cognition, in the dentate gyrus of aged rats, as many as 85 genes were differentially expressed between rats that had learned the Morris Water Maze, from those who had not [108]. In addition, it is also possible that the number of new neurons does not actually decline with aging, but rather, become more quiescent, perhaps because of a smaller vascular and/or neurogenic niche [107]. Thus, the etiology of these declining numbers suggest that the putative decreased trophic support combined with increased sensitivity to negative regulators, such as glutamate and cortisol [109], with age may lead to a suppression of neural stem cell proliferation and maturation [107,110]. Or perhaps in old animals, the rate of neural progenitor cell proliferation in the dentate gyrus is much lower than that in younger ones [111]. Alternatively, perhaps there is decreased survival of neural progenitor cells or change in neural differentiation [111]. 

Physical activity regulates neurogenesis in both the young adult and aging brain [102,112], although young runners had higher neurogenesis levels than the older [112,113] or middle-aged [114] runners. Consistent with the findings of Redila and Christie [75], who found that in rats, running is not correlated with granule cell dendritic complexity, others found that exercise restores the loss of intra-hippocampal connectivity [114,115], neurogenesis [105,112,114,116] and BDNF and TrkB levels [114] that accompanies sedentary aging [67]. Temporally, aging suppresses *in vitro* neural stem cell proliferation in mice beginning at six months of age [117]. A short period of voluntary wheel running in middle-aged female mice increased neural stem cell and progenitor cells in the subventricular zone [117]. Thus, Blackmore *et al*. [117] found that 12-month old mice lose approximately one-half of their neurogenic capacity because of normal age-related decline. It would appear, then, in sedentary mice, there is a 12-month age limit (~one-half their lifespan) beyond which the brain can no longer effectively maintain neural stem cell and progenitor cell viability. Exercise can effectively raise the neurogenic potential age limit to 18 months, as shown by the ability to recuperate neural stem cell proliferation levels after irradiation-induced suppression/neural stem cell death [117]. Consistently, exercise may increase neurotrophin expression to delay neurotrophin senescence [92]. However, only up to a point: In very aged mice (22 months of age), running exercise neither affected dentate gyrus neurogenesis nor angiogenesis [113,118], which may be a chronological extension of earlier findings in 18-month old mice [112]. Inconsistently, such late-onset exercise which begins relatively late in life, reverses the expression of many hippocampal genes changed by aging, such as by decreasing and increasing inflammatory and neurotrophic factor expression, respectively [119]. It is possible that the different mouse strains used between these two groups [118,119] accounted for the contradictory results.

Age-induced increases in inflammatory responses, as well as a decline in positive regulators of neurogenesis and angiogenesis (e.g., IGF-1, above) suggest that the circulation would also impact the former. Injections of plasma from old mice into young mice inhibited neurogenesis in the latter, indicating the presence of soluble factors in the aged blood that inhibit neurogenesis [120]. 

### The Duration of Running Exercise and the Age at Which an Animal Begins an Exercise Regimen Determines Whether Neurogenesis and Cell Survival Occurs

Obviously, the younger an animal is when an exercise regimen begins, the more likely it is that exercise will become a lifestyle or habit, rather than just simply a regimen. Neurogenesis, therefore, will occur in a dose-dependent manner. In younger animals, cell proliferation was dose-dependent with respect to running exercise: compared to those of sedentary controls, granule cell survival increased after both 14 and 21 days of running [85]; in contrast, granule cell proliferation increased after 12, but not after 19 days of running, suggesting that running accelerated the maturation of newly generated neurons [85]. In older animals, although only one week of running exercise has revealed neither proliferation nor neurogenesis in older adult rats [84], a 21-day bout of running exercise significantly increased neuronal proliferation in mice 18 months of age or older [117]. Such hippocampal cell proliferation was also observed in 18-month old female mice that were allowed running wheel access following group-housing-imposed stress [121]. Although such comparisons in exercise-induced neurogenesis between rats and mice may not be valid (above), such results nevertheless suggest that overall, running exercise reverses the age-dependent decline in neurogenesis in the mouse dentate gyrus [116].

## 6. Wnt as a Direct Actor in Neurogenesis and its Interaction with Other Signaling Pathways

As indicated above, Wnt signaling plays a significant role in the generation of adult hippocampal neurons. The convergence among multiple pathways, including that of Wnt, MAPK, PI-3K/Akt and PKA-cAMP to ultimately change gene expression [108] in the wake of physical activity hinges on exercise-induced increase in norepinephrine (and serotonin (5HT)) and subsequent release of BDNF (above, Figure 3).

### 6.1. In the Absence of Wnt Signaling

When Wnt signaling is off, β-catenin is associated with adherins and cadherins at the cell-cell junctions. Any β-catenin not associated with these proteins is rapidly degraded by proteosomes (above, Figure 2). Large multi-protein complexes will recruit β-catenin and at least three other proteins [34]: (i) GSK-3β, which will phosphorylate β-catenin, where the latter is subject to ubiquination in proteosomes, making it unstable; (ii) APC, which helps promote the degradation of β-catenin by increasing the affinity of the degradation complex for β-catenin (such as that carried out by GSK-3β); and (iii) the scaffolding, chaperonin-like protein, axin, which holds the protein complex (GSK-3β/APC/axin) together ([34], Figure 2).

### 6.2. In the Presence of Wnt Signaling

When Wnt signaling is on, Wnt binds to its fzl receptor, which is complexed with LDL co-receptor, LRP, thereby activating dsh1, which then inactivates GSK-3β in the degradation complex (GSK-3β/APC/axin) [122]. Another kinase, casein kinase I, also phosphorylates GSK-3β, thereby inactivating it. As a result, β-catenin is neither phosphorylated nor degraded and therefore; accumulates in the cytoplasm and nucleus (above, Figure 2). In the latter, β-catenin binds to LEF/TCF regulatory proteins, displaces a co-repressor, GROUCHO, and then acts as a co-activator to stimulate the transcription of the Wnt-responsive genes, one of which is *c-myc*.

#### Neurogenesis and Wnt Signaling

Adult hippocampal neural progenitors are self-sustaining, employing an autocrine baseline Wnt signaling loop within the neurogenic niche [41] (however, see below for evidence for astrocyte release of Wnt). Thus, adult hippocampal progenitor cells express Wnt3, whose Wnt3/β-catenin pathway is active in the neurogenic niche [42]. Moreover, over-expression of Wnt3 increases neurogenesis in these cells and inhibition of Wnt drastically decreases neurogenesis from these adult hippocampal progenitors [42]. In addition, Wnts, their fzl receptors and their co-receptors are activated as soon as 24 h after initial differentiation in a human hippocampal progenitor cell line [46]. Thus, β-catenin signaling increases neurogenesis in the subgranular zone; and inhibition of GSK-3β increases β-catenin signaling [55,123]. Recently, Valvezan and Klein [55] reviewed evidence that mutations in *Wnt1*, *3*, and *7* genes resulted in decreased or delayed hippocampal neurogenesis. Further, in dominant negative Wnt mutant rats, hippocampally dependent learning was impaired [39]. Located at the *NeuroD1* promotor are several LEF/TCF binding sites [81]. Transcription of the *NeuroD1* gene in the subgranular zone is increased as a result of Wnt3a signaling, in turn, leading to increased numbers of neuroprogenitors [55] (above and Figure 1).

Consistent with their role in initiating BDNF-mediated neurogenesis, in cultured hippocampal neurons, over-expression of fzl-1 receptors led to increased presynaptic clustering of bassoon [53] and in cultured astrocytes, Wnt3 shRNA led to increased expression of synapsin I [124]. In addition, treatment with Wnt3a [53] or Wnt7a [52] promoted presynaptic protein clustering, increased functional presynaptic recycling sites, and the rate of synaptic vesicle neurotransmitter release [52,53].

### 6.3. Wnt Signaling in Aging and Physical Activity

With general aging, there is a down-regulation of axonal growth, cytoskeletal assembly and transport, signaling, lipogenic uptake pathways and concomitant increase in immune/inflammatory lysosomal, protein/lipid degeneration, cholesterol transport, TGF and cAMP-mediated pathways [125]. In cognitively impaired aged rats, there is down-regulation of Wnt, insulin and its influences in lipid and glycogen pathways, and GPCR signaling [125]. However, recently, Miranda *et al*. [109] investigated the communication between neural progenitor cells and astrocytes. They applied survivin, a chromosomal passenger protein (*aka* Birc5), to neural progenitor cells. Age-associated changes in neural progenitor cell proliferation reveal an inverse correlation of a decrease in neural progenitor cell with age, indicating that astrocytes in the neurogenic niche initiate regulate changes in Wnt signaling via surviving regulation within neural progenitor cells [109]. That is, Wnts secreted from neighboring astrocytes regulate survivin expression and proliferation of adult neural progenitor cells [109].

Moreover, the secretion of Wnts by astrocytes regulates neural stem cell gene expression: in neural stem cells, a repressor complex, consisting of Sox2 and HDAC1 silences the *NeuroD1* gene promotor (above, [62]). Upon Wnt stimulation by astrocytes, β-catenin is activated accumulates in the nucleus, where it complexes with LEF/TCF, leading to transcription of the *NeuroD1* gene, leading to neurogenesis and maturation (above, [62]). Others have found that with age, NeuroD1 expression declines [59,61]. In neural stem cells, there are several L1 mobile elements that also contain multiple Sox/LEF sites and are normally silenced, but are activated following Wnt-mediated neurogenesis (above, [62]).

**Figure 4:**
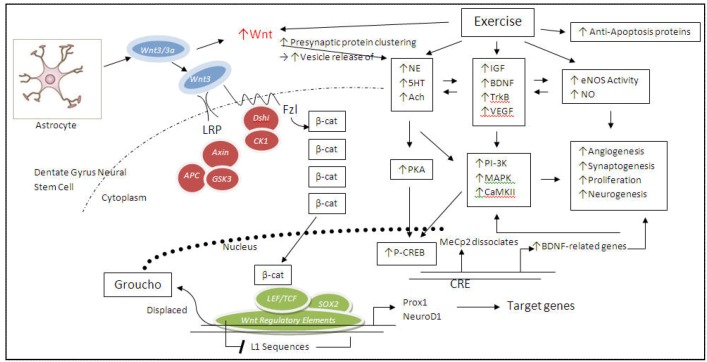
Exercise activates a wide variety of intracellular signal transduction pathways to promote neurogenesis of granule cells from neuroprogenitors in the dentate gyrus. Specifically applicable to neurogenesis, Wnt is released from neighboring astrocytes in a paracrine fashion, whereupon exercise increases Wnt signaling. Wnt binds its fzl receptor, complexed with LRP, and leading to the activation of Dshi, which, in turn, inactivates GSK3. The resulting accumulation of β-catenin (β-cat) in the cytoplasm and nucleus then binds to, and displaces, the gene regulatory proteins, LEF/TCF and Sox2 and the co-repressor, Groucho. β-Catenin then acts as a co-activator, and with transcription factors Prox1 and NeuroD1, stimulates the transcription of Wnt target genes. In addition, exercise also lifts the repressive MeCp2, thereby enhancing transcription. Normally, in the absence of exercise, Wnt3/3a is sent to the noncoding region of the granule cell, where L1 mobile elements are repressed during adult neurogenesis. Because L1 sequences contain the Wnt-regulatory element, the nearby genes can be indirectly up-regulated when the β-catenin/TCF-LEF complex is activated via Wnt signaling. In addition, exercise increases Wnt activity, leading to increased presynaptic protein and vesicle clustering, in turn leading to increased release of various neurotransmitters (norepinephrine, serotonin). Through a dizzying array of receptor and pathway cross-talk, these neurotransmitters, via GPCR signaling, can not only directly activate downstream PKA and subsequent transcription factor, CREB, thereby leading to the transcription of BDNF and related neurotrophic genes, but also activate other trophic factors (IGF-1, VEGF), thereby, in turn, activating a variety in intracellular signaling survival pathways (PI-3K, MAPK, CamKII), while simultaneously inhibiting apoptosis and inducing eNOS. Thus, both the Wnt regulatory element and the cAMP-response element (CRE) may participate (synergistically) to promote synaptogenesis, angiogenesis, proliferation, and neurogenesis.

Aging specifically compromises, whereas exercise increases, Wnt3 pathway signaling [126] and expression, thereby reversing the decline in neurogenesis brought on by age [124], as well as genes downstream of it [3,4] (Figure 4). In addition, as mentioned above, the study by Gogolla *et al*. [56] in which an enriched environment and Wnt7/7a application had the same effects on neurogenesis, it is possible that the running component of their living conditions was the crucial factor in eliciting neurogenesis [87]. The elegant studies by Okamoto *et al*. [124] have done much to contribute to our understanding of intercellular crosstalk between astrocytes and neural progenitor cells. *In vivo*, as age increases, astocytic Wnt3/3a expression and release decreases [126]. In addition, their *in vitro* experiments shed much light about the genetic regulation of Wnt-mediated neurogenesis. Their knockdowns of *fzl1* and β-catenin using siRNAs lead to a down-regulation of the TCF/LEF reporter expression in both young and aged neural stem cells, indicating that the expression of Wnt canonical signaling pathway intermediates was not impaired in aged neural stem cells. Moreover, lentivirus expressing Wnt3 shRNA in young and aged astrocytic cultures resulted in increased tubulin III and synapsin I expression, indicating that astrocytic Wnt3a causes a neurogenic effect on adult hippocampal neural stem cells in an age-dependent manner and that such cells are primed for increased growth and neurotransmitter release. Such specific function of what will eventually be the granule cell may be regulated by the *Prox1* promotor, which remains highly active throughout the maturation of the granule cell and may be responsible for specifying the neuronal phenotype [35]. Furthermore, Okamoto *et al*. [124] found that the *dcx* genes are among the L1 loci; specifically, the *dcx* promotor contains two L1 sequences regions with Wnt signaling regulatory sites. At the *NeuroD1* promotor, binding of acetylated histone A3, β-catenin, and CREB gradually decreases with age, indicating that the aging process controls the repressed chromatin state. Physical activity and Wnt, through increased release of norepinephrine, may lift this repression (see Section 4 above; Figure 4).

## 7. Summary and Conclusions

It is clear from the forgoing that although much is known about the effects of physical activity on neurogenesis, there is even more that is not known, specifically, the role of physical activity during the aging process. Several key studies and reviews reveal that in just the past five years, we have gained much understanding about the paracrine Wnt signaling between astrocytes and neural stem or progenitor cells. Although more than one transcriptional pathway is no doubt responsible for neurogenesis [127] and synaptogenesis (CREB, for example), much more work will have to be done to achieve the same level of understanding that we have with the CREB-mediated pathways. But, what happens during the aging process as it interacts with decreasing exercise? It is well known that animals tend to be less active as they age. Is it the aging process itself or lower physical activity levels or both that contribute to decreased Wnt signaling? Or, perhaps it is some other underlying pathology? Diabetic patients who do not exercise (enough) may experience impaired learning and memory because adult hippocampal neurogenesis from undifferentiated neural stem cells is severely curtailed [51], Figure 4). We know that less neuronal stimulation means less neurotransmitter and BDNF activity, and therefore, less transcriptional activity via CREB. But what about neural progenitor cell-derived Wnt *vs*. astrocytic Wnt signaling to neural stem cells? Which one prevails? And under what conditions? When during the animal’s lifetime? We reviewed studies showing that Wnt signaling decreases during aging, but is it physical activity *per se* that restores it? At the cellular level, exercise reverses the age-related decline in neurogenesis, but how does this happen? Clearly, more studies are needed to address these questions and possibly provide additional pharmacological therapeutic targets in aging and hippocampal pathology. 
